# Supplementary material for: Protocol for the development of a core indicator set for reporting burn wound infection in trials: ICon-B study
Source: BMJ Open. 2019 May 14;9(5):e026056. doi: 10.1136/bmjopen-2018-026056 (PMC6530370; doi:10.1136/bmjopen-2018-026056)
Supplement: Supplementary data [file bmjopen-2018-026056supp001.pdf]

Supplementary file: Example Search String for Systematic Review

1. exp burn/
2. burn*.tw.
3. scald*.tw.
4. thermal injur*.tw.
5. exp wound infection/
6. wound infection*.tw.
7. infection*.tw.
8. bacteria*.tw.
9. exp clinical trial/
10. clinical trial.tw
11. observ*.tw.
12. trial*.tw.
13. interven*.tw.
14. compar*.tw.
15. randomi*ed.tw.
16. 9 or 10 or 11 or 12 or 13 or 14 or 15
17. 1 or 2 or 3 or 4
18. Coxiella burnetii/
19. burnetii.tw.
20. 18 or 19
21. 17 not 20
22. 5 or 6 or 7 or 8
24. limit 23 to (human and english language and yr="2010 -Current")
