# Supplementary material for: Protocol for the development of a core indicator set for reporting burn wound infection in trials: ICon-B study
Source: BMJ Open. 2019 May 14;9(5):e026056. doi: 10.1136/bmjopen-2018-026056 (PMC6530370; doi:10.1136/bmjopen-2018-026056)
Supplement: Author's manuscript [file bmjopen-2018-026056.draft_revisions.pdf]

BMJ Open

BMJ Open is committed to open peer review. As part of this commitment we make the peer review history of every article we publish publicly available.

When an article is published we post the peer reviewers' comments and the authors' responses online. We also post the versions of the paper that were used during peer review. These are the versions that the peer review comments apply to.

The versions of the paper that follow are the versions that were submitted during the peer review process. They are not the versions of record or the final published versions. They should not be cited or distributed as the published version of this manuscript.

BMJ Open is an open access journal and the full, final, typeset and author-corrected version of record of the manuscript is available on our site with no access controls, subscription charges or pay-per-view fees (http://bmjopen.bmj.com).

If you have any questions on BMJ Open's open peer review process please email info.bmjopen@bmj.com

BMJ Open

Protocol for the development of a Core Indicator Set for reporting burn wound infection in trials: The ICon-B study

Journal:	*BMJ Open*
Manuscript ID	bmjopen-2018-026056
Article Type:	Protocol
Date Submitted by the Author:	15-Aug-2018

Complete List of Authors:	Davies, Anna; University of Bristol, Centre for Academic Child Health<br>Teare, Louise; Chelmsford Hospital, Department of Microbiology<br>Falder, Sian; Alder Hey Children's NHS Foundation Trust, Burns and Plastic Surgery<br>Coy, Karen; University Hospitals Bristol NHS Foundation Trust, Centre for Children's Burns Research<br>Dumville, Jo C.; Univ Manchester,<br>Collins, Declan; Chelsea and Westminster Hospital NHS Foundation Trust, Plastic Surgery Department; Imperial College London Department of Surgery and Cancer<br>Moore, Luke; Chelsea and Westminster Hospital NHS Foundation Trust, Department of Microbiology; Imperial College London, National Institute for Health Research Health Protection Research Unit<br>Dheansa, Baljit; Queen Victoria Hospital NHS Foundation Trust, Department of Plastic Surgery and Burns<br>Jenkins, Toby; University of Bath, Department of Chemistry<br>Booth, Simon; University of Brighton, School of Pharmacy and Biomolecular Sciences<br>Agha, Riaz; Royal Free Hospital NHS Foundation Trust, Department of Plastic Surgery<br>Shah, Mamta; Royal Manchester Children's Hospital, Department of Plastic Surgery<br>Young, Amber; University Hospitals Bristol NHS Foundation Trust, Paediatric Anaesthesia; University of Bristol, Canynge Hall

Keywords:	burn wound infection, systematic review, Delphi survey, diagnostic indicators

SCHOLARONE™
Manuscripts

Protocol for the development of a Core Indicator Set for reporting burn wound infection in trials: The ICon-B study

Authors: Davies, A.K.[1], Teare, L.[2], Falder, S[3]., Coy, K.[4], Dumville, J.[5], Collins, D.[6], Moore, L.S.P.[7], Dheansa, B.[8], Jenkins, A.T.A.[9] , Booth, S.P.[10], Agha, R.A.[11], Shah, M.[12] Young, A.E.R. [4, 13]

Corresponding Author:

[1]Anna Davies

Centre for Academic Child Health

University of Bristol

1-5 Whiteladies Road

Bristol BS8 1NU

Tel: 0117 428 3103

anna.davies@bristol.ac.uk

[2] Department of Microbiology, Broomfield Hospital, Chelmsford CM1 7ET, UK

louise.teare@meht.nhs.uk

[3] Plastic Surgery Department, Alder Hey Children's NHS Foundation Trust, Eaton Road, Liverpool, L12 2AP, UK sian.falder@alderhey.nhs.uk

[4] Centre for Children's Burns Research, Bristol Royal Hospital for Children, Bristol, BS2 8BJ, UK karen.coy@uhbristol.nhs.uk

[5] Division of Nursing, Midwifery and Social Work, School of Health Sciences, Faculty of Biology, Medicine and Health, University of Manchester, Manchester Academic Health Science Centre, Manchester, M13 9PL, UK jo.dumville@manchester.ac.uk

For peer review only

For peer review only - http://bmjopen.bmj.com/site/about/guidelines.xhtml

[6] a) Plastic Surgery Department, Chelsea and Westminster Hospital, London, SW10 9NH, and b) Department of Surgery and Cancer, Imperial College, London SW7 2AZ, UK declan.collins@chelwest.nhs.uk

[7]a) Department of Microbiology, Chelsea and Westminster NHS Foundation Trust, London SW10 9NH, and b) National Institute for Health Research Health Protection Research Unit in Healthcare Associated Infections and Antimicrobial Resistance, Imperial College London, London, W12 0NN, UK luke.moore@nhs.net

[8]Baljit Dheansa, Department of Plastic Surgery and Burns, Queen Victoria Hospital, East Grinstead, Sussex, RH19 3DZ,UK b.dheansa@nhs.net

[9]Toby Jenkins, Department of Chemistry, University of Bath, Bath, BA2 2NU, UK a.t.a.jenkins@bath.ac.uk

[10]Simon Booth, Research Fellow, School of Pharmacy and Biomolecular Sciences, University of Brighton, Brighton, BN2 4GJ, UK simon.booth@nhs.net

[11]Riaz Agha, Department of Plastic Surgery, Royal Free Hospital NHS Foundation Trust, London, NW3 2QG, UK mail@riazagha.com

[12]Mamta Shah, Department of Plastic Surgery, Royal Manchester Children's Hospital, M13 9WL, UK mamta.shah@mft.nhs.uk

[13]Amber Young, Population Health Sciences, University of Bristol, Canynge Hall, 39 Whatley Road, Bristol, BS8 2PS, UK amber.young1@nhs.net

Word Count: 3575

For peer review only - http://bmjopen.bmj.com/site/about/guidelines.xhtml

ABSTRACT

Introduction: Systematic reviews of high quality randomised controlled trials are necessary to identify effective interventions to detect, treat or prevent burn wound infection (BWI). Evidence synthesis requires that BWI is reported in a consistent manner. Cochrane reviews investigating interventions for burns report that the indicators used to diagnose BWI are variable or not described, indicating a need to standardise reporting. BWI is diagnosed by clinician judgement, informed by patient-reported symptoms, clinical signs, serum markers of inflammation and bacteria in the wound. With no objective method to identify BWI, consensus statements have been developed. Their use is limited by inclusion of indicators infrequently seen in patients with BWI, and reliance on tests not universally undertaken in UK practice. Indicators for reporting BWI should be important for diagnosis, frequently observed in patients with BWI, and assessed as part of routine healthcare. A minimum (Core) set of indictors of BWI, reported consistently, will facilitate evidence synthesis and support clinical decision-making.

Aims: The Infection Consensus in Burns (ICon-B) study aims to identify a Core Indicator Set for reporting the diagnosis of BWI in research studies.

Methods and Analysis: 1. Evidence review: A systematic review of indicators used in trials and observational studies reporting BWI outcomes to identify a long-list of candidate indicators, 2. Refinement of the long-list into a smaller set of survey questions with an expert steering group, 3. A two-round Delphi survey with 100 multidisciplinary expert stakeholders, to achieve consensus on a short-list of indicators, 4. A consensus meeting with expert stakeholders to agree the BWI Core Indicator Set.

Ethics and Dissemination: Participants will be recruited through professional bodies, such that NHS HRA ethical approval is not needed. The Core Indicator Set will be disseminated

For peer review only - http://bmjopen.bmj.com/site/about/guidelines.xhtml

through peer-reviewed publication, co-production with journal editors, research funders and professional bodies, and presentation at national conferences.

Strengths and weaknesses of the study:

- This protocol was co-produced by a multidisciplinary panel with expertise in diagnosing burn wound infection.
- A systematic review will be undertaken to identify all reported diagnostic indicators of burn wound infection to inform the candidate indicator list.
- We will work closely with multi-disciplinary stakeholders who will be identified through professional bodies to participate in the Delphi survey, facilitate dissemination and optimise research impact.
- A UK focus balances limiting the application of the Core Indicator Set with identifying a pragmatic, useable set of indicators for research in the NHS.

For peer review only

For peer review only - http://bmjopen.bmj.com/site/about/guidelines.xhtml

INTRODUCTION

Over 140,000 patients in England and Wales required specialised burn care between 2010 and 2017(1). Data indicates that the majority of burns treated in the UK are small in size, with a median of between 1% and 2.5% of total body surface area (TBSA) affected(2) (3). Patients with burns are susceptible to burn wound infection (BWI) due to the loss of the skin's barrier to microbes and the impact of the burn on the immune system(4). It is estimated that approximately 10-20% of patients with burns will develop BWI before healing(4). These patients may experience delayed wound healing(5), require re-grafting(6) and can develop sepsis if left untreated. To identify the most effective interventions to prevent, detect and treat BWI, systematic synthesis of evidence from high-quality randomised controlled trials (RCTs) is needed(7).

Recent systematic reviews have found that while RCTs typically report BWI as a single outcome, there is considerable variation in the indicators used to inform the diagnosis(8-10). In a Cochrane review of 36 RCTs evaluating the effectiveness of antibiotic prophylaxis in burn patients(8), five defined BWI as the presence of more than 10^5 organisms per gram of tissue, 10 used positive wound swab culture, 11 used signs and symptoms consistent with BWI and six studies did not define the diagnostic indicators used. In another Cochrane Review assessing topical therapy for facial burns(10), of 3 studies reporting BWI, 3 different indicators were used for diagnosis; one study used swab cultures, one used qualitative assessment of exudate and cellulitis, and the third diagnosed BWI if there was chondritis. If the reporting of BWI is based on varying diagnostic indicators, or if the diagnostic indicators used are not reported, the validity of the findings is compromised, limiting the ability of the review to provide evidence to inform clinical decision making.

For peer review only - http://bmjopen.bmj.com/site/about/guidelines.xhtml

One explanation for this heterogeneity in the reported indicators used to diagnose BWI in research trials is that clinical diagnosis of BWI is challenging; there is no accepted objective method for diagnosing BWI and diagnosis relies on clinical judgement. Clinical diagnosis is subjective(4), and based on several indicators of infection. These include patient reported symptoms, clinical wound assessment, systemic signs, the presence of bacteria in a wound, retrospective response to antibiotics and non-specific serum markers of inflammation. Patients typically need to have only some of these symptoms and signs to be diagnosed with, and treated for, BWI. Quantitative bacterial counts from swab cultures are acknowledged as providing only supportive information, as all wounds are colonised with bacteria within 48 hours of injury (11, 12). Diagnosis is further complicated by the fact that many signs used in diagnosis of BWI, such as pyrexia and tachycardia, are not specific to BWI, and are present as part of the normal systemic inflammatory response (SIR) to burn wound injury(13).

In the absence of an objective method for diagnosing BWI, consensus statements have been developed with expert stakeholders to support diagnosis of burn wound infection and sepsis. These include the American Burns Association (ABA(14)), the Center for Disease Control (CDC(15)), and the European Wound Management Association (EWMA(16)) consensus statements. However, there are limitations of these statements that preclude their application clinically and in research. The ABA and CDC statements use information gathered from several potential indicators of BWI to result in a composite outcome of BWI. Composite outcomes of BWI have not, and cannot be, tested for diagnostic accuracy due to the absence of an objective reference standard. There are also practical limitations relating to the indicators used to underpin diagnosis. Both the ABA and CDC consensus statements rely on the use of wound biopsy for diagnosis of wound infection. Biopsies are infrequently carried out in the UK because they are costly, invasive, painful and require anaesthesia(17) . The evidence base for the use of quantitative microbiology, such as wound biopsies, for

For peer review only - http://bmjopen.bmj.com/site/about/guidelines.xhtml

diagnosing clinically relevant BWI is limited(11, 17). Limitations of the EWMA consensus include a lack of reference to systemic signs of infection, and inclusion of indicators that, while highly sensitive, are infrequently seen in patients with BWI, for example Ecthyma Gangrenosum. For these reasons these previously developed consensus statements may have limited usefulness for identifying or reporting BWI in UK practice.

Until an evidence-based, objective, reference standard for determining the presence of BWI is available, there is a need to develop a consistent approach for reporting the indicators used to inform diagnosis in research. Agreement regarding a minimum (core) set of symptoms, signs and laboratory tests considered to be important in determining the presence of BWI, frequently seen in patients diagnosed with BWI, and assessed as part of normal care, will allow standardisation of reporting of BWI in trials.

For peer review only

Aim

The Infection Consensus in Burns study (ICon-B) aims to achieve the development of a Core Indicator Set to standardise the reporting of symptoms, signs and laboratory tests used for diagnosing burn wound infection in research studies.

METHOD:

Phase 1: Systematic review: Identifying potential candidate indicators of BWI

A systematic review will be undertaken to identify potential indicators of BWI used in current research to evaluate the effects of interventions on BWI outcomes (PROSPERO Registration CRD42018096647).

For peer review only - http://bmjopen.bmj.com/site/about/guidelines.xhtml

Identifying relevant studies: Pre-defined inclusion and exclusion criteria will be used to identify relevant studies. Included studies will be any peer-reviewed, published study or study protocol reporting an evaluation of any intervention, using a randomised or non-randomised trial design or an observational methodology. They will be included if they report the effect of an intervention or treatment for patients with burns where a BWI outcome is reported in the study methods, results or discussion. Study participants will be any patient with a cutaneous burn injury of any cause and any size. Exclusion criteria are indicated in table 1.

INSERT TABLE 1 HERE

Search: A search string will be developed based on above criteria and will comprise synonyms of burns, wound infection, interventions and trials or observational studies (see supplementary file for example search). It will be applied to five databases: Ovid Medline, Ovid Embase, Cinahl, Cochrane Register of Clinical Trials, Cochrane Register of Protocols. The search will be limited to studies published after January 1st 2010 to ensure that current knowledge and practice regarding BWI is identified. Studies written in languages other than English will be excluded since there is no funding available for translation.

Selection: The inclusion and exclusion criteria will be applied by one researcher in two stages. These will first be applied to titles and abstracts identified from the search, and then to full-texts of studies retained following title and abstract screening. At each stage a second researcher will review 20% of the screened studies for inclusion, and reasons for exclusion, to ensure reliability of study selection.

Data extraction: A pro-forma will be developed to conduct systematic data extraction with fields relating to study design, intervention/s tested, participant age, gender, burn size, mechanism of injury. Verbatim reports of each indicator for the diagnosis of BWI will be extracted, and where provided, the methods for assessing the indicator (e.g. bacterial samples

For peer review only - http://bmjopen.bmj.com/site/about/guidelines.xhtml

taken using swab), and any standard parameters used to determine presence of infection (e.g. body temperature >38°c; bacterial count 10^5 microbes per gram of tissue). One reviewer will conduct all data extraction with a second reviewer extracting data from 20% of studies, to ensure reliability of data extraction. Disagreements will be discussed, and a third reviewer will facilitate decision-making where required. Second-reviewer checking of data extraction will be continued until consistency is achieved.

Data analysis and reporting: the extracted indicators will be tabulated and examined. Similar indicators will be grouped. A second reviewer will review groupings, and disagreements will be resolved through discussion. Where previously described tools (e.g. ABA, CDC and EWMA consensus statements) have been used to diagnose presence of infection, this will be reported. Their incorporated indicators will be deconstructed for inclusion in the long-list of candidate indicators.

Phase 2: Reduction of the long-list of indicators into a shorter list

The long-list of BWI indicators identified in the described systematic review will be reduced by removing duplicates. A steering group will be convened comprising clinicians (doctors, nurses, microbiologists) and researchers with an interest in burns. A face to face steering group meeting will be held to review the long-list of indicators. Each single indicator or grouping will be assigned a label. The labels will be refined through discussion, to achieve a clear definition for each. The labels will be categorised into patient-reported symptoms, clinically-observed signs or laboratory tests.

Phase 3: Achieving consensus about BWI indicators using an online Delphi survey

A modified Delphi method will be used, comprising two questionnaire rounds and a final consensus meeting.

For peer review only - http://bmjopen.bmj.com/site/about/guidelines.xhtml

An online questionnaire will be developed using REDCap(18) to enable distribution of the questionnaire to a wide stakeholder audience. Before use, the questionnaire will be piloted with a small number of stakeholders from all relevant groups, to check understanding and ease of use, as well as data capture and feedback processes.

Sample: There is no agreed standard sample size for conducting a Delphi survey(19). Up to 100 stakeholders representing the different staff involved in BWI diagnosis will be recruited, including consultant surgeons working in burn care, junior medical and nursing staff with at least 3 months experience of burn care, clinical microbiologists working in burn services, and General Practitioners and Emergency Department staff who report that they see at least one patient with suspected BWI per month. Stakeholders will be recruited through mass email to the memberships of appropriate professional groups (e.g. the British Burns Association, Care of Burns in Scotland, Royal College of General Practitioners/Emergency Medicine, Association for Clinical Biochemistry and Laboratory Medicine, Healthcare Infection Society, British Society of Antimicrobial Chemotherapy). We will aim to ensure an even distribution of respondents between groups.

Ethics: Since recruitment will take place outside of NHS organisations and through professional bodies, Health Research Authority approval is not required.

Questionnaire round 1: Participants completing the survey will be asked to rate each of the BWI indicator items on three scales:

i. The importance of the indicator for reporting presence of burn wound infection in research.

e.g. 'How important is this indicator in diagnosing burn wound infection?'
Participants will indicate their responses to each indicator on a 9-point Likert-type scale ranging from 1- 9 with written anchors to indicate 1-3 (not at all important), 4-6 (important

For peer review only - http://bmjopen.bmj.com/site/about/guidelines.xhtml

but not essential), and 7-9 (very important). Participants may indicate that they have no view about the indicator where appropriate.

ii. The frequency with which the indicator is seen in patients with burn wound infection. *e.g. 'How frequently do you see this symptom/sign in patients diagnosed with burn wound infection?'*

Participants will respond to each indicator on a nine-point Likert-type scale ranging from 1-9, with written anchors to indicate 1-3 (never seen)', 4-6 (sometimes seen) and 7-9 (Seen in most patients diagnosed with a burn wound infection). Participants may indicate that they have no view about the indicator where appropriate.

iii. Whether the indicator is or can be assessed as part of normal practice for a patient with burns e.g. '*Do you regularly use this indicator to establish the presence or absence of burn wound infection in your day to day practice?'*

Participants will respond by indicating whether they would normally assess the indicator as a Yes or No. Participants may indicate that they have no view about the indicator where appropriate.

At the end of the survey participants will be invited to state any further indicators not included that they believe are important for consideration in the second survey round.

Calculating consensus about items

The distribution of ratings for each indicator relating to i) importance and ii) frequency with which the indicator is seen in patients with BWI will be graphed and examined. Percentages will be calculated for the number of participants rating the item 1-3 (not at all important/ never seen), 4-6 (important but not essential/ sometimes seen) and 7-9 (very important/ seen in most patients diagnosed with a wound infection). Additionally, the percentage will be

For peer review only - http://bmjopen.bmj.com/site/about/guidelines.xhtml

calculated of participants indicating that they iii) assess the indicator as part of normal practice (% stating yes/ no).

Indicators carried through to the second round of the Delphi:

Indicators will only be carried through to the second round of the Delphi where ≥75% of the sample indicate that they assess it as part of normal practice. If this criterion is met, indicators will then be assessed as to whether there is consensus that the item is important and/or frequently seen. Definitions of consensus vary between Delphi surveys; it is accepted that there is a need to balance being so inclusive that a parsimonious core indicator set is not achieved, with using such stringent criteria that important indicators are missed(20). In several recent core outcome set protocols and studies using Delphi methodology, a threshold of 70% of the sample agreeing that a criterion is important has been used(21-23). In the current study a more stringent threshold of ≥75% of the sample falling within the bottom (1-3) or top third of the scale (7-9) of the importance and frequency scales will indicate consensus about the importance/frequency with which an indicator is seen, only where a maximum of 15% of the sample rates it in the opposing third.

Therefore, indicators to be carried forward to the second round of the survey are those where ≥75% of the sample rate it as 7-9 on the Likert-type scale, with ≤15% of the sample rating it as 1-3. Indicators will also be carried through to the second round where there is *no consensus* about their importance or frequency i.e. fewer than ≥ 75% of the sample rate it in either the top or bottom third of the scale.

Items will be removed from the indicator list and not carried through to the second round if:

1. ≤ 74% report that they do not assess it as part of their normal practice, **OR**

For peer review only - http://bmjopen.bmj.com/site/about/guidelines.xhtml

2. There is consensus that the indicator is unimportant, **OR** infrequently seen in patients with BWI, whereby ≥75% of the sample rate it as 1-3, and ≤15% rate it as 7-9 on these scales.

Round 2: Participants completing the first questionnaire will receive a second survey, comprising the indicators retained from the previous round, using the same item and response format. Additional indicators provided by the respondents in the first round will be incorporated into the second survey round.

In addition, participants will receive tailored feedback showing their own and other stakeholders' responses to each indicator, including the central tendency (e.g. mean, median or mode) and distribution of ratings for each item (e.g. standard deviation or Inter-quartile range). Methods for establishing the most effective means for presenting this information will be piloted prior to survey development. It is not anticipated that feedback will be provided indicating item scores according to stakeholder group. However, if there is disparity in responses between groups of stakeholders, this decision will be considered by the study steering group.

Participants will be invited to re-visit and if desired, alter, their rating for each item and to re-state their ratings for each indicator in relation to i) importance, ii) frequency, iii) whether it is used for diagnosis.

Indicators carried through to the stakeholder meeting

Indicators will be taken to the stakeholder meeting if they meet the proceed criteria described above. More stringent criteria may be applied if it is apparent that this process has not reduced the potential indicator list to an acceptable number.

For peer review only - http://bmjopen.bmj.com/site/about/guidelines.xhtml

Phase 4: Stakeholder consensus meeting

All survey participants will be invited to a consensus meeting. During this meeting, data will be presented regarding counts/frequency, or central tendency, and the distribution of scores for each indicator relating to i) whether it is assessed, ii) importance, and iii) frequency with which it is observed in patients considered to have BWI. Following review of all indicators, participants will be invited to prioritise indicators for inclusion using anonymous electronic voting software. A final list of indicators will be agreed and a method for reporting the core indicator set in research will be discussed.

Public and patient involvement

Patients and members of the public were not involved in the development of this protocol, since diagnosis of BWI is made by clinicians.

DISCUSSION

Heterogeneous methods and reporting limits the extent to which evidence synthesis using data from randomised controlled trials can provide valid information about the effectiveness of treatments. Systematic literature reviews of treatments for patients with burns demonstrate that there is variation in the indicators used to diagnose BWI in trials, limiting the conclusions that can be drawn about the effectiveness of treatments. In the absence of an objective means of diagnosing BWI, agreement about a set of the most important (core) symptoms, signs and laboratory tests (indicators) for diagnosing BWI will standardise research reporting. This will result in evidence syntheses that provide more valid information about treatment effects. This protocol describes a systematic and pragmatic multiphase

For peer review only - http://bmjopen.bmj.com/site/about/guidelines.xhtml

approach to developing a Core Indicator Set for BWI, taking account of expert views from the multi-disciplinary team involved in BWI diagnosis.

The Delphi methodology is one method to develop consensus among experts on a given topic. It has been used previously to establish consensus between experts about core outcomes to be assessed in various healthcare domains, and has been found to improve consistency of outcome reporting between studies(24). This methodology has also been used to agree key diagnostic criteria for clinical conditions for which there is no objective diagnostic method, including for bone and joint infections in children(25) and hepatic and renal cyst infection(26). It is important to note that the proposed methodology for developing the Core Indicator Set is not intended to identify those indicators that are most predictive of BWI diagnosis. Rather, its purpose is to identify which indicators multidisciplinary stakeholders agree are important and applicable to routine patient care in the UK, to standardise the reporting of BWI in research studies. It should also be noted that while the resulting list of indicators is a recommended minimum that should be reported, it will not preclude the reporting of data relating to other indicators relevant to BWI diagnosis, should researchers wish to do so.

The proposed work addresses the practical limitations of previously developed consensus-based criteria for diagnosing BWI (ABA(27), CDC(15) and EWMA(28)). These consensus statements provide a limited basis for a Core Indicator Set since they include tests that are not currently widely used in NHS practice (e.g. wound biopsy; ABA, CDC), and prioritise signs of BWI that are infrequently seen in patients with burns (e.g. ecthyma gangrenosum; EWMA(28)). Our proposed study aims to take account not only of whether the indicator is viewed as important in the diagnosis of BWI, it will also ensure that the indicator is likely to be seen, and can be assessed in patients with burns. This will ensure that the resulting Core

For peer review only - http://bmjopen.bmj.com/site/about/guidelines.xhtml

Indicator Set is applicable and relevant to care in the UK, and that data about the indicators can be readily captured for reporting in research studies.

A limitation of the current work is that the literature search to identify current indicators used to diagnose BWI is limited to studies published in English, and that the Delphi survey will only be conducted in the United Kingdom. Nonetheless, a global perspective is brought through the identification of international studies published in English. The practical applicability of the core indicator set to NHS practice is balanced against the potential that other indicators than those identified by a UK stakeholder group may be considered of equal or greater importance and usefulness by an international audience.

Impact and dissemination:

To ensure effective uptake of the Core Indicator Set by burn care researchers, early buy-in is essential. By engaging and working closely with the multidisciplinary stakeholder steering group to develop the questions for the Delphi study, and the active participation in the Delphi survey by a wide group of relevant clinical stakeholders, we hope to ensure future uptake of the resulting Core Indicator Set by its intended audience. We aim to publish the Core Indicator Set in a burns-specific journal to reach potential users. Further, we will engage with editors of appropriate journals, and the Cochrane Collaboration Wounds Group, to facilitate its take-up for trial reporting. Dissemination of the Core Indicator Set and the development work underpinning it described here will be carried out through presentations at appropriate wound- and burn-related conferences.

Conclusion

The ICon-B study aims to identify a core set of indicators to standardise the reporting of BWI in research studies. The indicators will be those considered to be important for identifying the

For peer review only - http://bmjopen.bmj.com/site/about/guidelines.xhtml

presence of burn wound infection, frequently observed in patients with BWI and assessed as part of usual care. A systematic approach to identifying candidate signs, symptoms and tests will be undertaken. The participation of end-user stakeholders to reach consensus about their inclusion will optimise buy-in and impact for the final Core Indicator Set. This will result in consistent recording of data about burn wound infection across studies and will allow the accumulation and comparison of evidence to identify the most effective treatments for patients with burns in relation to BWI.

For peer review only

REFERENCES

1. International Burn Injury Database (iBID) 2018 [Available from: www.ibidb.org. Accessed 01 August 2018.

2. Stylianou N, Buchan I, Dunn KW. A review of the international Burn Injury Database (iBID) for England and Wales: descriptive analysis of burn injuries 2003-2011. BMJ Open. 2015;5(2):e006184.

3. Battle CE, Evans V, James K, Guy K, Whitley J, Evans PA. Epidemiology of burns and scalds in children presenting to the emergency department of a regional burns unit: a 7-year retrospective study. Burns Trauma. 2016;4:19.

4. Church D, Elsayed S, Reid O, Winston B, Lindsay R. Burn Wound Infections. Clin Microbiol Rev. 2006;19(2):403-34.

5. Singer AJ, McClain SA. Persistent wound infection delays epidermal maturation and increases scarring in thermal burns. Wound Repair Regen. 2002;10(6):372-7.

For peer review only - http://bmjopen.bmj.com/site/about/guidelines.xhtml

6. Posluszny JA, Conrad P, Halerz M, Shankar R, Gamelli RL. Surgical burn wound infections and their clinical implications. Journal of Burn Care and Research. 2011;32(2):324-33.

7. Evans D. Hierarchy of evidence: a framework for ranking evidence evaluating healthcare interventions. J Clin Nurs. 2003;12(1):77-84.

8. Barajas-Nava LA, Lopez-Alcalde J, Roque i Figuls M, Sola I, Bonfill Cosp X. Antibiotic prophylaxis for preventing burn wound infection. The Cochrane database of systematic reviews. 2013;6:Cd008738.

9. Avni T, Levcovich A, Ad-El DD, Leibovici L, Paul M. Prophylactic antibiotics for burns patients: systematic review and meta-analysis. BMJ (Clinical research ed). 2010;340:c241.

10. Hoogewerf CJ, Van Baar ME, Hop MJ, Nieuwenhuis MK, Oen IM, Middelkoop E. Topical treatment for facial burns. The Cochrane database of systematic reviews. 2013(1):CD008058.

11. Halstead FD, Lee KC, Kwei J, Dretzke J, Oppenheim BA, Moiemen NS. A systematic review of quantitative burn wound microbiology in the management of burns patients. Burns : journal of the International Society for Burn Injuries. 2018;44(1):39-56.

12. Erol S, Altoparlak U, Akcay MN, Celebi F, Parlak M. Changes of microbial flora and wound colonization in burned patients. Burns : journal of the International Society for Burn Injuries. 2004;30(4):357-61.

13. Norbury W, Herndon DN, Tanksley J, Jeschke MG, Finnerty CC. Infection in Burns. Surg Infect (Larchmt). 2016;17(2):250-5.

14. Greenhalgh DG, Saffle JR, Holmes JHt, Gamelli RL, Palmieri TL, Horton JW, et al. American Burn Association consensus conference to define sepsis and infection in burns.

For peer review only

For peer review only - http://bmjopen.bmj.com/site/about/guidelines.xhtml

Journal of burn care & research : official publication of the American Burn Association. 2007;28(6):776-90.

15. Horan TC, Andrus M, Dudeck MA. CDC/NHSN surveillance definition of health care-associated infection and criteria for specific types of infections in the acute care setting. Am J Infect Control. 2008;36(5):309-32.

16. Cutting K.F. WRJ, Mahoney P., Harding K.G. Clinical Identification of Wound Infection: a Delphi Approach. London: MEP; 2005 2005.

17. Edwards-Jones V, Greenwood JE, Manchester Burns Research G. What's new in burn microbiology? James Laing Memorial Prize Essay 2000. Burns: Journal of the International Society for Burn Injuries. 2003;29(1):15-24.

18. Harris PA, Taylor R, Thielke R, Payne J, Gonzalez N, Conde JG. Research electronic data capture (REDCap)-A metadata-driven methodology and workflow process for providing translational research informatics support. J Biomed Inform. 2009;42(2):377-81.

19. Akins RB, Tolson H, Cole BR. Stability of response characteristics of a Delphi panel: application of bootstrap data expansion. BMC Med Res Methodol. 2005;5:37.

20. Kirkham JJ, Davis K, Altman DG, Blazeby JM, Clarke M, Tunis S, et al. Core Outcome Set-STAndards for Development: The COS-STAD recommendations. Plos Med. 2017;14(11):e1002447.

21. Meher S, Cuthbert A, Kirkham JJ, Williamson P, Abalos E, Aflaifel N, et al. Core outcome sets for prevention and treatment of postpartum haemorrhage: an international Delphi consensus study. BJOG. 2018.

22. Young A, Brookes S, Rumsey N, Blazeby J. Agreement on what to measure in randomised controlled trials in burn care: study protocol for the development of a core outcome set. Bmj Open. 2017;7(6).

For peer review only - http://bmjopen.bmj.com/site/about/guidelines.xhtml

23. Allin BSR, Hall NJ, Ross AR, Marven SS, Kurinczuk JJ, Knight M, et al. Development of a gastroschisis core outcome set. Arch Dis Child Fetal Neonatal Ed. 2018.

24. Kirkham JJ, Clarke M, Williamson PR. A methodological approach for assessing the uptake of core outcome sets using ClinicalTrials.gov: findings from a review of randomised controlled trials of rheumatoid arthritis. Bmj-Brit Med J. 2017;357.

25. Mitha A, Boulyana M, Hue V, Pruvost I, Martinot A, European French-speaking expert g, et al. Consensus in diagnostic definitions for bone or joint infections in children by a Delphi method with European French-speaking experts. Acta Paediatr. 2012;101(8):e350-6.

26. Lantinga MA, Darding AJ, de Sevaux RG, Alam A, Bleeker-Rovers CP, Bobot M, et al. International Multi-Specialty Delphi Survey: Identification of Diagnostic Criteria for Hepatic and Renal Cyst Infection. Nephron. 2016;134(4):205-14.

27. Greenhalgh DG, Saffle JR, Holmes JH, Gamelli RL, Palmieri TL, Horton JW, et al. American burn association consensus conference to define sepsis and infection in burns. Journal of Burn Care and Research. 2007;28(6):776-90.

28. Cutting K.F. WRJ, Mahoney P., Harding K.G. Clinical identification of wound infection: a Delphi approach. EWMA Position document: Identifying criteria for wound infection [Internet]. 2005 17/07/2018:[6-9 pp.]. Available from: http://old.ewma.org/fileadmin/user_upload/EWMA/pdf/Position_Documents/2005__Wound_Infection_/English_pos_doc_final.pdf.

Author contributions:

Anna Davies and Amber Young drafted the manuscript for this study. Detailed input into the design of the proposed work and comprehensive editorial comments were given by all other authors.

For peer review only - http://bmjopen.bmj.com/site/about/guidelines.xhtml

Funding statement: This work was supported by the Medical Research Council Grant number: MR/No06496/1. Development of an infection detecting wound dressing.

Competing interests: No competing interests

Table 1: Inclusion and exclusion criteria for systematic review

Inclusion criteria	Exclusion criteria
• Trial or observational study assessing the impact of an intervention for patients with burns<br>• Reports a BWI outcome in the methods, results or discussion	• Not written in English<br>• Not exclusively about burns (e.g. trauma population)<br>• Non-human subjects<br>• Laboratory based, not carried out in clinical setting e.g. exudate or blood samples from humans tested within laboratory<br>• Does not report a BWI outcome, or reports other infection (e.g. UTI, CLABSI)<br>• Abstract, systematic review or commentary paper<br>• Not a trial or observational study reporting an intervention to treat burn injuries

For peer review only

For peer review only - http://bmjopen.bmj.com/site/about/guidelines.xhtml

Supplementary file: Example Search String for Systematic Review

1. exp burn/
2. burn*.tw.
3. scald*.tw.
4. thermal injur*.tw.
5. exp wound infection/
6. wound infection*.tw.
7. infection*.tw.
8. bacteria*.tw.
9. exp clinical trial/
10. clinical trial.tw
11. observ*.tw.
12. trial*.tw.
13. interven*.tw.
14. compar*.tw.
15. randomi*ed.tw.
16. 9 or 10 or 11 or 12 or 13 or 14 or 15
17. 1 or 2 or 3 or 4
18. Coxiella burnetii/
19. burnetii.tw.
20. 18 or 19
21. 17 not 20
22. 5 or 6 or 7 or 8
24. limit 23 to (human and english language and yr="2010 -Current")

For peer review only

BMJ Open

Protocol for the development of a Core Indicator Set for reporting burn wound infection in trials: The ICon-B study

Journal:	*BMJ Open*
Manuscript ID	bmjopen-2018-026056.R1
Article Type:	Protocol
Date Submitted by the Author:	21-Feb-2019
Complete List of Authors:	Davies, Anna; University of Bristol, Centre for Academic Child Health<br>Teare, Louise; Chelmsford Hospital, Department of Microbiology<br>Falder, Sian; Alder Hey Children's NHS Foundation Trust, Burns and Plastic Surgery<br>Coy, Karen; University Hospitals Bristol NHS Foundation Trust, Centre for Children's Burns Research<br>Dumville, Jo C.; Univ Manchester,<br>Collins, Declan; Chelsea and Westminster Hospital NHS Foundation Trust, Plastic Surgery Department; Imperial College London Department of Surgery and Cancer<br>Moore, Luke; Chelsea and Westminster Hospital NHS Foundation Trust, Department of Microbiology; Imperial College London, National Institute for Health Research Health Protection Research Unit<br>Dheansa, Baljit; Queen Victoria Hospital NHS Foundation Trust, Department of Plastic Surgery and Burns<br>Jenkins, Toby; University of Bath, Department of Chemistry<br>Booth, Simon; University of Brighton, School of Pharmacy and Biomolecular Sciences<br>Agha, Riaz; Royal Free Hospital NHS Foundation Trust, Department of Plastic Surgery<br>Shah, Mamta; Royal Manchester Children's Hospital, Department of Plastic Surgery<br>Marlow, Karen; Alder Hey Children's NHS Foundation Trust, Burns and Plastic Surgery<br>Young, Amber; University Hospitals Bristol NHS Foundation Trust, Paediatric Anaesthesia; University of Bristol, Canynge Hall
**Primary Subject Heading**:	Infectious diseases
Secondary Subject Heading:	Research methods
Keywords:	burn wound infection, systematic review, Delphi survey, diagnostic indicators, burns

Protocol for the development of a Core Indicator Set for reporting burn wound infection in trials: The ICon-B study

Authors: Davies, A.K.[1], Teare, L.[2], Falder, S[3]., Coy, K.[4], Dumville, J.[5], Collins, D.[6], Moore, L.S.P.[7], Dheansa, B.[8], Jenkins, A.T.A.[9] , Booth, S.P.[10], Agha, R.A.[11], Shah, M[12]., Marlow, K. [13] Young, A.E.R. [4, 14]

Corresponding Author:

[1]Anna Davies

Centre for Academic Child Health

University of Bristol

1-5 Whiteladies Road

Bristol BS8 1NU

Tel: 0117 428 3103

anna.davies@bristol.ac.uk

[2] Department of Microbiology, Broomfield Hospital, Chelmsford CM1 7ET, UK

louise.teare@meht.nhs.uk

[3] Plastic Surgery Department, Alder Hey Children's NHS Foundation Trust, Eaton Road, Liverpool, L12 2AP, UK sian.falder@alderhey.nhs.uk

[4] Centre for Children's Burns Research, Bristol Royal Hospital for Children, Bristol, BS2 8BJ, UK karen.coy@uhbristol.nhs.uk;

[5] Division of Nursing, Midwifery and Social Work, School of Health Sciences, Faculty of Biology, Medicine and Health, University of Manchester, Manchester Academic Health Science Centre, Manchester, M13 9PL, UK jo.dumville@manchester.ac.uk

For peer review only

For peer review only - http://bmjopen.bmj.com/site/about/guidelines.xhtml

[6] a) Plastic Surgery Department, Chelsea and Westminster Hospital, London, SW10 9NH, and b) Department of Surgery and Cancer, Imperial College, London SW7 2AZ, UK declan.collins@chelwest.nhs.uk

[7]a) Department of Microbiology, Chelsea and Westminster NHS Foundation Trust, London SW10 9NH, and b) National Institute for Health Research Health Protection Research Unit in Healthcare Associated Infections and Antimicrobial Resistance, Imperial College London, London, W12 0NN, UK luke.moore@nhs.net

[8]Baljit Dheansa, Department of Plastic Surgery and Burns, Queen Victoria Hospital, East Grinstead, Sussex, RH19 3DZ,UK b.dheansa@nhs.net

[9]Toby Jenkins, Department of Chemistry, University of Bath, Bath, BA2 2NU, UK a.t.a.jenkins@bath.ac.uk

[10]Simon Booth, Research Fellow, School of Pharmacy and Biomolecular Sciences, University of Brighton, Brighton, BN2 4GJ, UK simon.booth@nhs.net

[11]Riaz Agha, Department of Plastic Surgery, Royal Free Hospital NHS Foundation Trust, London, NW3 2QG, UK mail@riazagha.com

[12]Mamta Shah, Department of Plastic Surgery, Royal Manchester Children's Hospital, M13 9WL, UK mamta.shah@mft.nhs.uk

[13] Karen Marlow, Alder Hey Children's NHS Foundation Trust, Eaton Road, Liverpool, L12 2AP, U, karen.marlow@alderhey.nhs.uk

[14]Amber Young, Population Health Sciences, University of Bristol, Canynge Hall, 39 Whatley Road, Bristol, BS8 2PS, UK amber.young1@nhs.net

Word Count: 3575

For peer review only - http://bmjopen.bmj.com/site/about/guidelines.xhtml

ABSTRACT

Introduction: Systematic reviews of high quality randomised controlled trials are necessary to identify effective interventions to impact burn wound infection outcomes (BWI). Evidence synthesis requires that BWI is reported in a consistent manner. Cochrane reviews investigating interventions for burns report that the indicators used to diagnose BWI are variable or not described, indicating a need to standardise reporting. BWI is complex and diagnosed by clinician judgement, informed by patient-reported symptoms, clinical signs, serum markers of inflammation and bacteria in the wound. Indicators for reporting BWI should be important for diagnosis, frequently observed in patients with BWI, and assessed as part of routine healthcare. A minimum (core) set of indictors of BWI, reported consistently, will facilitate evidence synthesis and support clinical decision-making.

Aims: The Infection Consensus in Burns (ICon-B) study aims to identify a Core Indicator Set for reporting the diagnosis of BWI in research studies.

Methods and Analysis: 1. Evidence review: A systematic review of indicators used in trials and observational studies reporting BWI outcomes to identify a long-list of candidate indicators, 2. Refinement of the long-list into a smaller set of survey questions with an expert steering group, 3. A two-round Delphi survey with 100 multidisciplinary expert stakeholders, to achieve consensus on a short-list of indicators, 4. A consensus meeting with expert stakeholders to agree the BWI Core Indicator Set.

Ethics and Dissemination: Participants will be recruited through professional bodies, such that NHS HRA ethical approval is not needed. The Core Indicator Set will be disseminated through peer-reviewed publication, co-production with journal editors, research funders and professional bodies, and presentation at national conferences.

For peer review only - http://bmjopen.bmj.com/site/about/guidelines.xhtml

For peer review only

Strengths and weaknesses of the study:

- This protocol was co-produced by a multidisciplinary panel with expertise in diagnosing burn wound infection.
- A systematic review will be undertaken to identify all reported diagnostic indicators of burn wound infection to inform the candidate indicator list.
- We will work closely with multi-disciplinary stakeholders who will be identified through professional bodies to participate in the Delphi survey, facilitate dissemination and optimise research impact.
- A UK focus balances limiting the application of the Core Indicator Set with identifying a pragmatic, useable set of indicators for research in the NHS.

For peer review only

For peer review only - http://bmjopen.bmj.com/site/about/guidelines.xhtml

INTRODUCTION

Over 140,000 patients in England and Wales required specialised burn care between 2010 and 2017(1). Data indicates that the majority of burns treated in the UK are small in size, with a median of between 1% and 2.5% of total body surface area (TBSA) affected(2) (3). Patients with burns are susceptible to burn wound infection (BWI) due to the loss of the skin's barrier to microbes and the impact of the burn on the immune system(4). It is estimated that approximately 10-20% of patients with burns will develop BWI before healing(4). These patients may experience delayed wound healing(5), require re-grafting(6) and can develop sepsis if left untreated. To identify the most effective interventions to prevent, detect and treat BWI, systematic synthesis of evidence from high-quality randomised controlled trials (RCTs) is needed(7).

Recent systematic reviews have found that while RCTs typically report BWI as a single outcome, there is considerable variation in the indicators used to inform the diagnosis(8-10). In a Cochrane review of 36 RCTs evaluating the effectiveness of antibiotic prophylaxis in burn patients(8), five defined BWI as the presence of more than 10^5 organisms per gram of tissue, 10 used positive wound swab culture, 11 used signs and symptoms consistent with BWI and six studies did not define the diagnostic indicators used. In another Cochrane Review assessing topical therapy for facial burns(10), of 3 studies reporting BWI, 3 different indicators were used for diagnosis; one study used swab cultures, one used qualitative assessment of exudate and cellulitis, and the third diagnosed BWI if there was chondritis. If the reporting of BWI is based on varying diagnostic indicators, or if the diagnostic indicators used are not reported, the validity of the findings is compromised, limiting the ability of the review to provide evidence to inform clinical decision making.

For peer review only - http://bmjopen.bmj.com/site/about/guidelines.xhtml

One explanation for this heterogeneity in the reported indicators used to diagnose BWI in research trials is that clinical diagnosis of BWI is challenging; there is no accepted objective method for diagnosing BWI and diagnosis relies on clinical judgement. Clinical diagnosis is subjective(4), and based on several indicators of infection. These include patient reported symptoms, clinical wound assessment, systemic signs, the presence of bacteria in a wound, retrospective response to antibiotics and non-specific serum markers of inflammation. Patients typically need to have only some of these symptoms and signs to be diagnosed with, and treated for, BWI. Quantitative bacterial counts from swab cultures are acknowledged as providing only supportive information, as all wounds are colonised with bacteria within 48 hours of injury (11, 12). Diagnosis is further complicated by the fact that many signs used in diagnosis of BWI, such as pyrexia and tachycardia, are not specific to BWI, and are present as part of the normal systemic inflammatory response (SIR) to burn wound injury(13).

In the absence of an objective method for diagnosing BWI, consensus statements have been developed with expert stakeholders to support diagnosis of burn wound infection and sepsis. These include the American Burns Association (ABA(14)), the Center for Disease Control (CDC(15)), and the European Wound Management Association (EWMA(16)) consensus statements. However, there are limitations of these statements that preclude their application clinically and in research. The ABA and CDC statements use information gathered from several potential indicators of BWI to result in a composite outcome of BWI. Composite outcomes of BWI have not, and cannot be, tested for diagnostic accuracy due to the absence of an objective reference standard. There are also practical limitations relating to the indicators used to underpin diagnosis. Both the ABA and CDC consensus statements rely on the use of wound biopsy for diagnosis of wound infection. Biopsies are infrequently carried out in the UK because they are costly, invasive, painful and require anaesthesia(17) . The evidence base for the use of quantitative microbiology, such as wound biopsies, for

For peer review only - http://bmjopen.bmj.com/site/about/guidelines.xhtml

diagnosing clinically relevant BWI is limited(11, 17). Limitations of the EWMA consensus include a lack of reference to systemic signs of infection, and inclusion of indicators that, while highly sensitive, are infrequently seen in patients with BWI, for example Ecthyma Gangrenosum. For these reasons these previously developed consensus statements may have limited usefulness for identifying or reporting BWI in UK practice.

Until an evidence-based, objective, reference standard for determining the presence of BWI is available, there is a need to develop a consistent approach for reporting the indicators used to inform diagnosis in research. Agreement regarding a minimum (core) set of symptoms, signs and laboratory tests considered to be important in determining the presence of BWI, frequently seen in patients diagnosed with BWI, and assessed as part of normal care, will allow standardisation of reporting of BWI in trials.

For peer review only

Aim

The Infection Consensus in Burns study (ICon-B) aims to achieve the development of a Core Indicator Set to standardise the reporting of symptoms, signs and laboratory tests used for diagnosing burn wound infection in research studies.

METHOD:

Phase 1: Systematic review: Identifying potential candidate indicators of BWI

A systematic review will be undertaken to identify potential indicators of BWI used in current research to evaluate the effects of interventions on BWI outcomes (PROSPERO Registration CRD42018096647).

For peer review only - http://bmjopen.bmj.com/site/about/guidelines.xhtml

Identifying relevant studies: Pre-defined inclusion and exclusion criteria will be used to identify relevant studies. Included studies will be any peer-reviewed, published study or study protocol reporting an evaluation of any intervention, using a randomised or non-randomised trial design or an observational methodology. They will be included if they report the effect of an intervention or treatment for patients with burns where a BWI outcome is reported in the study methods, results or discussion. Study participants will be any patient with a cutaneous burn injury of any cause and any size. Exclusion criteria are indicated in table 1.

INSERT TABLE 1 HERE

Search: A search string will be developed based on above criteria and will comprise synonyms of burns, wound infection, interventions and trials or observational studies (see supplementary file for example search). It will be applied to five databases: Ovid Medline, Ovid Embase, Cinahl, Cochrane Register of Clinical Trials, Cochrane Register of Protocols. The search will be limited to studies published after January 1st 2010 to ensure that current knowledge and practice regarding BWI is identified. Studies written in languages other than English will be excluded since there is no funding available for translation.

Selection: The inclusion and exclusion criteria will be applied by one researcher in two stages. These will first be applied to titles and abstracts identified from the search, and then to full-texts of studies retained following title and abstract screening. At each stage a second researcher will review 20% of the screened studies for inclusion, and reasons for exclusion, to ensure reliability of study selection.

Data extraction: A pro-forma will be developed to conduct systematic data extraction with fields relating to study design, intervention/s tested, participant age, gender, burn size, mechanism of injury. Verbatim reports of each indicator for the diagnosis of BWI will be extracted, and where provided, the methods for assessing the indicator (e.g. bacterial samples

For peer review only - http://bmjopen.bmj.com/site/about/guidelines.xhtml

taken using swab), and any standard parameters used to determine presence of infection (e.g. body temperature >38°c; bacterial count 10^5 microbes per gram of tissue). One reviewer will conduct all data extraction with a second reviewer extracting data from 20% of studies, to ensure reliability of data extraction. Disagreements will be discussed, and a third reviewer will facilitate decision-making where required. Second-reviewer checking of data extraction will be continued until consistency is achieved.

Data analysis and reporting: the extracted indicators will be tabulated and examined. Similar indicators will be grouped. A second reviewer will review groupings, and disagreements will be resolved through discussion. Where previously described tools (e.g. ABA, CDC and EWMA consensus statements) have been used to diagnose presence of infection, this will be reported. Their incorporated indicators will be deconstructed for inclusion in the long-list of candidate indicators.

Phase 2: Reduction of the long-list of indicators into a shorter list

The long-list of BWI indicators identified in the described systematic review will be reduced by removing duplicates. A steering group will be convened comprising clinicians (doctors, nurses, microbiologists) and researchers with an interest in burns. A face to face steering group meeting will be held to review the long-list of indicators. Each single indicator or grouping will be assigned a label. The labels will be refined through discussion, to achieve a clear definition for each. The labels will be categorised into patient-reported symptoms, clinically-observed signs or laboratory tests.

Phase 3: Achieving consensus about BWI indicators using an online Delphi survey

A modified Delphi method will be used, comprising two questionnaire rounds and a final consensus meeting.

For peer review only

An online questionnaire will be developed using REDCap(18) to enable distribution of the questionnaire to a wide stakeholder audience. Before use, the questionnaire will be piloted with a small number of stakeholders from all relevant groups, to check understanding and ease of use, as well as data capture and feedback processes.

Sample: There is no agreed standard sample size for conducting a Delphi survey(19). Up to 100 stakeholders representing the different staff involved in BWI diagnosis will be recruited, including consultant surgeons working in burn care, junior medical and nursing staff with at least 3 months experience of burn care, clinical microbiologists working in burn services, and General Practitioners and Emergency Department staff who report that they see at least one patient with suspected BWI per month. Stakeholders will be recruited through mass email to the memberships of appropriate professional groups (e.g. the British Burns Association, Care of Burns in Scotland, Royal College of General Practitioners/Emergency Medicine, Association for Clinical Biochemistry and Laboratory Medicine, Healthcare Infection Society, British Society of Antimicrobial Chemotherapy). We will aim to ensure an even distribution of respondents between groups.

Ethics and dissemination: Recruitment of stakeholder participants will take place outside of NHS organisations and through professional bodies. Therefore, Health Research Authority and research ethics approvals are not required to conduct the survey.

The findings of the systematic review will be presented at relevant burns- and wound-research related conferences and written up for publication. The findings of the review will directly inform the items used in the Delphi survey and stakeholder consensus event. Following establishment of a set of core reporting criteria, we will disseminate the findings as a peer reviewed journal article, and through appropriate professional conferences. We will

For peer review only - http://bmjopen.bmj.com/site/about/guidelines.xhtml

work with journal editors from relevant journals, and will disseminate our findings to relevant funders to increase awareness of the core reporting criteria.

Questionnaire round 1: Participants completing the survey will be asked to rate each of the BWI indicator items on three scales:

i. The importance of the indicator for reporting presence of burn wound infection in research.

e.g. '*How important is this indicator in diagnosing burn wound infection?'*
Participants will indicate their responses to each indicator on a 9-point Likert-type scale ranging from 1- 9 with written anchors to indicate 1-3 (not at all important), 4-6 (important but not essential), and 7-9 (very important). Participants may indicate that they have no view about the indicator where appropriate.

ii. The frequency with which the indicator is seen in patients with burn wound infection. *e.g. 'How frequently do you see this symptom/sign in patients diagnosed with burn wound infection?'*

Participants will respond to each indicator on a nine-point Likert-type scale ranging from 1-9, with written anchors to indicate 1-3 (never seen)', 4-6 (sometimes seen) and 7-9 (Seen in most patients diagnosed with a burn wound infection). Participants may indicate that they have no view about the indicator where appropriate.

iii. Whether the indicator is or can be assessed as part of normal practice for a patient with burns e.g. '*Do you regularly use this indicator to establish the presence or absence of burn wound infection in your day to day practice?'*

Participants will respond by indicating whether they would normally assess the indicator as a Yes or No. Participants may indicate that they have no view about the indicator where appropriate.

For peer review only - http://bmjopen.bmj.com/site/about/guidelines.xhtml

At the end of the survey participants will be invited to state any further indicators not included that they believe are important for consideration in the second survey round.

Calculating consensus about items

The distribution of ratings for each indicator relating to i) importance and ii) frequency with which the indicator is seen in patients with BWI will be graphed and examined. Percentages will be calculated for the number of participants rating the item 1-3 (not at all important/ never seen), 4-6 (important but not essential/ sometimes seen) and 7-9 (very important/ seen in most patients diagnosed with a wound infection). Additionally, the percentage will be calculated of participants indicating that they iii) assess the indicator as part of normal practice (% stating yes/ no).

Indicators carried through to the second round of the Delphi:

Indicators will only be carried through to the second round of the Delphi where ≥75% of the sample indicate that they assess it as part of normal practice. If this criterion is met, indicators will then be assessed as to whether there is consensus that the item is important and/or frequently seen. Definitions of consensus vary between Delphi surveys; it is accepted that there is a need to balance being so inclusive that a parsimonious core indicator set is not achieved, with using such stringent criteria that important indicators are missed(20). In several recent core outcome set protocols and studies using Delphi methodology, a threshold of 70% of the sample agreeing that a criterion is important has been used(21-23). In the current study a more stringent threshold of ≥75% of the sample falling within the bottom (1-3) or top third of the scale (7-9) of the importance and frequency scales will indicate

For peer review only - http://bmjopen.bmj.com/site/about/guidelines.xhtml

consensus about the importance/frequency with which an indicator is seen, only where a maximum of 15% of the sample rates it in the opposing third.

Therefore, indicators to be carried forward to the second round of the survey are those where ≥75% of the sample rate it as 7-9 on the Likert-type scale, with ≤15% of the sample rating it as 1-3. Indicators will also be carried through to the second round where there is *no consensus* about their importance or frequency i.e. fewer than ≥ 75% of the sample rate it in either the top or bottom third of the scale.

Items will be removed from the indicator list and not carried through to the second round if:

1. ≤ 74% report that they do not assess it as part of their normal practice, **OR**
2. There is consensus that the indicator is unimportant, **OR** infrequently seen in patients with BWI, whereby ≥75% of the sample rate it as 1-3, and ≤15% rate it as 7-9 on these scales.

Round 2: Participants completing the first questionnaire will receive a second survey, comprising the indicators retained from the previous round, using the same item and response format. Additional indicators provided by the respondents in the first round will be incorporated into the second survey round.

In addition, participants will receive tailored feedback showing their own and other stakeholders' responses to each indicator, including the central tendency (e.g. mean, median or mode) and distribution of ratings for each item (e.g. standard deviation or Inter-quartile range). Methods for establishing the most effective means for presenting this information will be piloted prior to survey development. It is not anticipated that feedback will be provided indicating item scores according to stakeholder group. However, if there is disparity in

For peer review only - http://bmjopen.bmj.com/site/about/guidelines.xhtml

responses between groups of stakeholders, this decision will be considered by the study steering group.

Participants will be invited to re-visit and if desired, alter, their rating for each item and to re-state their ratings for each indicator in relation to i) importance, ii) frequency, iii) whether it is used for diagnosis.

Indicators carried through to the stakeholder meeting

Indicators will be taken to the stakeholder meeting if they meet the proceed criteria described above. More stringent criteria may be applied if it is apparent that this process has not reduced the potential indicator list to an acceptable number.

Phase 4: Stakeholder consensus meeting

All survey participants will be invited to a consensus meeting. During this meeting, data will be presented regarding counts/frequency, or central tendency, and the distribution of scores for each indicator relating to i) whether it is assessed, ii) importance, and iii) frequency with which it is observed in patients considered to have BWI. Following review of all indicators, participants will be invited to prioritise indicators for inclusion using anonymous electronic voting software. A final list of indicators will be agreed and a method for reporting the core indicator set in research will be discussed.

Public and patient involvement

Patients and members of the public were not involved in the development of this protocol, since diagnosis of BWI is made by clinicians.

For peer review only - http://bmjopen.bmj.com/site/about/guidelines.xhtml

DISCUSSION

Heterogeneous methods and reporting limits the extent to which evidence synthesis using data from randomised controlled trials can provide valid information about the effectiveness of treatments. Systematic literature reviews of treatments for patients with burns demonstrate that there is variation in the indicators used to diagnose BWI in trials, limiting the conclusions that can be drawn about the effectiveness of treatments. In the absence of an objective means of diagnosing BWI, agreement about a set of the most important (core) symptoms, signs and laboratory tests (indicators) for diagnosing BWI will standardise research reporting. This will result in evidence syntheses that provide more valid information about treatment effects. This protocol describes a systematic and pragmatic multiphase approach to developing a Core Indicator Set for BWI, taking account of expert views from the multi-disciplinary team involved in BWI diagnosis.

The Delphi methodology is one method to develop consensus among experts on a given topic. It has been used previously to establish consensus between experts about core outcomes to be assessed in various healthcare domains, and has been found to improve consistency of outcome reporting between studies(24). This methodology has also been used to agree key diagnostic criteria for clinical conditions for which there is no objective diagnostic method, including for bone and joint infections in children(25) and hepatic and renal cyst infection(26). It is important to note that the proposed methodology for developing the Core Indicator Set is not intended to identify those indicators that are most predictive of BWI diagnosis. Rather, its purpose is to identify which indicators multidisciplinary stakeholders agree are important and applicable to routine patient care in the UK, to standardise the reporting of BWI in research studies. It should also be noted that while the

For peer review only - http://bmjopen.bmj.com/site/about/guidelines.xhtml

resulting list of indicators is a recommended minimum that should be reported, it will not preclude the reporting of data relating to other indicators relevant to BWI diagnosis, should researchers wish to do so.

The proposed work addresses the practical limitations of previously developed consensus-based criteria for diagnosing BWI (ABA(27), CDC(15) and EWMA(28)). These consensus statements provide a limited basis for a Core Indicator Set since they include tests that are not currently widely used in NHS practice (e.g. wound biopsy; ABA, CDC), and prioritise signs of BWI that are infrequently seen in patients with burns (e.g. ecthyma gangrenosum; EWMA(28)). Our proposed study aims to take account not only of whether the indicator is viewed as important in the diagnosis of BWI, it will also ensure that the indicator is likely to be seen, and can be assessed in patients with burns. This will ensure that the resulting Core Indicator Set is applicable and relevant to care in the UK, and that data about the indicators can be readily captured for reporting in research studies.

A limitation of the current work is that the literature search to identify current indicators used to diagnose BWI is limited to studies published in English, and that the Delphi survey will only be conducted in the United Kingdom. Nonetheless, a global perspective is brought through the identification of international studies published in English. The practical applicability of the core indicator set to NHS practice is balanced against the potential that other indicators than those identified by a UK stakeholder group may be considered of equal or greater importance and usefulness by an international audience.

Impact and dissemination:

To ensure effective uptake of the Core Indicator Set by burn care researchers, early buy-in is essential. By engaging and working closely with the multidisciplinary stakeholder steering group to develop the questions for the Delphi study, and the active participation in the Delphi

For peer review only - http://bmjopen.bmj.com/site/about/guidelines.xhtml

survey by a wide group of relevant clinical stakeholders, we hope to ensure future uptake of the resulting Core Indicator Set by its intended audience. We aim to publish the Core Indicator Set in a burns-specific journal to reach potential users. Further, we will engage with editors of appropriate journals, and the Cochrane Collaboration Wounds Group, to facilitate its take-up for trial reporting. Dissemination of the Core Indicator Set and the development work underpinning it described here will be carried out through presentations at appropriate wound- and burn-related conferences.

Conclusion

The ICon-B study aims to identify a core set of indicators to standardise the reporting of BWI in research studies. The indicators will be those considered to be important for identifying the presence of burn wound infection, frequently observed in patients with BWI and assessed as part of usual care. A systematic approach to identifying candidate signs, symptoms and tests will be undertaken. The participation of end-user stakeholders to reach consensus about their inclusion will optimise buy-in and impact for the final Core Indicator Set. This will result in consistent recording of data about burn wound infection across studies and will allow the accumulation and comparison of evidence to identify the most effective treatments for patients with burns in relation to BWI.

Contributors: AEY and AD wrote the paper and conceived the project. LT, AJ and LM provided microbiological expertise. SF, KC, DC, BJ, SB, RA, MS, KM provided clinical and nursing expertise relating to wound infection diagnosis. JD provided methodological expertise. All authors critically reviewed and contributed to the manuscript. All authors and have read and approved it.

For peer review only - http://bmjopen.bmj.com/site/about/guidelines.xhtml

Competing Interests: No authors report conflicts of interest that have influenced what was submitted, readers could perceive to have influenced, or that give the appearance of potentially influencing, what was written in the submitted work.

REFERENCES

1. International Burn Injury Database (iBID) 2018 [Available from: www.ibidb.org. Accessed 01 August 2018.

2. Stylianou N, Buchan I, Dunn KW. A review of the international Burn Injury Database (iBID) for England and Wales: descriptive analysis of burn injuries 2003-2011. BMJ Open. 2015;5(2):e006184.

3. Battle CE, Evans V, James K, Guy K, Whitley J, Evans PA. Epidemiology of burns and scalds in children presenting to the emergency department of a regional burns unit: a 7-year retrospective study. Burns Trauma. 2016;4:19.

4. Church D, Elsayed S, Reid O, Winston B, Lindsay R. Burn Wound Infections. Clin Microbiol Rev. 2006;19(2):403-34.

5. Singer AJ, McClain SA. Persistent wound infection delays epidermal maturation and increases scarring in thermal burns. Wound Repair Regen. 2002;10(6):372-7.

6. Posluszny JA, Conrad P, Halerz M, Shankar R, Gamelli RL. Surgical burn wound infections and their clinical implications. Journal of Burn Care and Research. 2011;32(2):324-33.

7. Evans D. Hierarchy of evidence: a framework for ranking evidence evaluating healthcare interventions. J Clin Nurs. 2003;12(1):77-84.

For peer review only - http://bmjopen.bmj.com/site/about/guidelines.xhtml

8. Barajas-Nava LA, Lopez-Alcalde J, Roque i Figuls M, Sola I, Bonfill Cosp X. Antibiotic prophylaxis for preventing burn wound infection. The Cochrane database of systematic reviews. 2013;6:Cd008738.

9. Avni T, Levcovich A, Ad-El DD, Leibovici L, Paul M. Prophylactic antibiotics for burns patients: systematic review and meta-analysis. BMJ (Clinical research ed). 2010;340:c241.

10. Hoogewerf CJ, Van Baar ME, Hop MJ, Nieuwenhuis MK, Oen IM, Middelkoop E. Topical treatment for facial burns. The Cochrane database of systematic reviews. 2013(1):CD008058.

11. Halstead FD, Lee KC, Kwei J, Dretzke J, Oppenheim BA, Moiemen NS. A systematic review of quantitative burn wound microbiology in the management of burns patients. Burns : journal of the International Society for Burn Injuries. 2018;44(1):39-56.

12. Erol S, Altoparlak U, Akcay MN, Celebi F, Parlak M. Changes of microbial flora and wound colonization in burned patients. Burns : journal of the International Society for Burn Injuries. 2004;30(4):357-61.

13. Norbury W, Herndon DN, Tanksley J, Jeschke MG, Finnerty CC. Infection in Burns. Surg Infect (Larchmt). 2016;17(2):250-5.

14. Greenhalgh DG, Saffle JR, Holmes JHt, Gamelli RL, Palmieri TL, Horton JW, et al. American Burn Association consensus conference to define sepsis and infection in burns. Journal of burn care & research : official publication of the American Burn Association. 2007;28(6):776-90.

15. Horan TC, Andrus M, Dudeck MA. CDC/NHSN surveillance definition of health care-associated infection and criteria for specific types of infections in the acute care setting. Am J Infect Control. 2008;36(5):309-32.

For peer review only

16. Cutting K.F. WRJ, Mahoney P., Harding K.G. Clinical Identification of Wound Infection: a Delphi Approach. London: MEP; 2005 2005.

17. Edwards-Jones V, Greenwood JE, Manchester Burns Research G. What's new in burn microbiology? James Laing Memorial Prize Essay 2000. Burns: Journal of the International Society for Burn Injuries. 2003;29(1):15-24.

18. Harris PA, Taylor R, Thielke R, Payne J, Gonzalez N, Conde JG. Research electronic data capture (REDCap)-A metadata-driven methodology and workflow process for providing translational research informatics support. J Biomed Inform. 2009;42(2):377-81.

19. Akins RB, Tolson H, Cole BR. Stability of response characteristics of a Delphi panel: application of bootstrap data expansion. BMC Med Res Methodol. 2005;5:37.

20. Kirkham JJ, Davis K, Altman DG, Blazeby JM, Clarke M, Tunis S, et al. Core Outcome Set-STAndards for Development: The COS-STAD recommendations. Plos Med. 2017;14(11):e1002447.

21. Meher S, Cuthbert A, Kirkham JJ, Williamson P, Abalos E, Aflaifel N, et al. Core outcome sets for prevention and treatment of postpartum haemorrhage: an international Delphi consensus study. BJOG. 2018.

22. Young A, Brookes S, Rumsey N, Blazeby J. Agreement on what to measure in randomised controlled trials in burn care: study protocol for the development of a core outcome set. Bmj Open. 2017;7(6).

23. Allin BSR, Hall NJ, Ross AR, Marven SS, Kurinczuk JJ, Knight M, et al. Development of a gastroschisis core outcome set. Arch Dis Child Fetal Neonatal Ed. 2018.

24. Kirkham JJ, Clarke M, Williamson PR. A methodological approach for assessing the uptake of core outcome sets using ClinicalTrials.gov: findings from a review of randomised controlled trials of rheumatoid arthritis. Bmj-Brit Med J. 2017;357.

For peer review only

For peer review only - http://bmjopen.bmj.com/site/about/guidelines.xhtml

25. Mitha A, Boulyana M, Hue V, Pruvost I, Martinot A, European French-speaking expert g, et al. Consensus in diagnostic definitions for bone or joint infections in children by a Delphi method with European French-speaking experts. Acta Paediatr. 2012;101(8):e350-6.

26. Lantinga MA, Darding AJ, de Sevaux RG, Alam A, Bleeker-Rovers CP, Bobot M, et al. International Multi-Specialty Delphi Survey: Identification of Diagnostic Criteria for Hepatic and Renal Cyst Infection. Nephron. 2016;134(4):205-14.

27. Greenhalgh DG, Saffle JR, Holmes JH, Gamelli RL, Palmieri TL, Horton JW, et al. American burn association consensus conference to define sepsis and infection in burns. Journal of Burn Care and Research. 2007;28(6):776-90.

28. Cutting K.F. WRJ, Mahoney P., Harding K.G. Clinical identification of wound infection: a Delphi approach. EWMA Position document: Identifying criteria for wound infection [Internet]. 2005 17/07/2018:[6-9 pp.]. Available from: http://old.ewma.org/fileadmin/user_upload/EWMA/pdf/Position_Documents/2005__Wound_Infection_/English_pos_doc_final.pdf.

Funding statement: This work was supported by the Medical Research Council Grant number: MR/No06496/1. Development of an infection detecting wound dressing.

For peer review only

Table 1: Inclusion and exclusion criteria for systematic review

**Inclusion criteria**	**Exclusion criteria**
• Trial or observational study assessing the impact of an intervention for patients with burns<br>• Reports a BWI outcome in the methods, results or discussion	• Not written in English<br>• Not exclusively about burns (e.g. trauma population)<br>• Non-human subjects<br>• Laboratory based, not carried out in clinical setting e.g. exudate or blood samples from humans tested within laboratory<br>• Does not report a BWI outcome<br>• Abstract, systematic review or commentary paper<br>• Not a trial or observational study reporting an intervention to treat burn injuries

For peer review only

For peer review only - http://bmjopen.bmj.com/site/about/guidelines.xhtml

Supplementary file: Example Search String for Systematic Review

1. exp burn/
2. burn*.tw.
3. scald*.tw.
4. thermal injur*.tw.
5. exp wound infection/
6. wound infection*.tw.
7. infection*.tw.
8. bacteria*.tw.
9. exp clinical trial/
10. clinical trial.tw
11. observ*.tw.
12. trial*.tw.
13. interven*.tw.
14. compar*.tw.
15. randomi*ed.tw.
16. 9 or 10 or 11 or 12 or 13 or 14 or 15
17. 1 or 2 or 3 or 4
18. Coxiella burnetii/
19. burnetii.tw.
20. 18 or 19
21. 17 not 20
22. 5 or 6 or 7 or 8
24. limit 23 to (human and english language and yr="2010 -Current")

For peer review only
